# Improving chairside ergonomic behavior in postgraduate dental students through a structured interprofessional education module: a quasi-experimental study

**DOI:** 10.3389/fpubh.2026.1919438

**Published:** 2026-07-24

**Authors:** S. Meenakshi, N. Raghunath, Amitabh Kishor Dwivedi

**Affiliations:** 1 Department of Prosthodontics, JSS Dental College & Hospital, JSS Academy of Higher Education & Research, Mysore, Karnataka, India; 2 Department of Orthodontics, JSS Dental College & Hospital, JSS Academy of Higher Education & Research, Mysore, Karnataka, India; 3 Department of Occupational Therapy, JSS Academy of Higher Education & Research, Mysore, Karnataka, India

**Keywords:** dental ergonomics, interprofessional education, MEND tool, musculoskeletal disorders, postgraduate dental students, quasi-experimental, work-related injury prevention

## Abstract

**Background:**

Inadequate ergonomic methods in dentistry put practitioners under disproportionate biomechanical strain, which increases the risk of musculoskeletal problems over the course of a working career. Because they carry out intricate, drawn-out chairside operations without further assistance, clinical postgraduate dentistry students constitute a high-risk population. This study assessed whether postgraduate dentistry students’ ergonomic knowledge, attitudes, and chairside behavior might be quantitatively improved by an organized interprofessional education (IPE) program.

**Methods:**

A quasi-experimental pre–post design was used. Prosthodontics, Periodontics, Oral Surgery, Conservative Dentistry, and Endodontics were the five clinical departments where all 36 postgraduate dental students were enrolled. Departments without direct patient-care duties were not included. The six-week course included chairside stretching exercises, environmental modification advice, interactive discussion, and didactic instruction. At weeks two and six, unannounced photos were used to grade ergonomic behavior using the validated MEND (Meenakshi’s Ergonomic Needs for Dentistry) assessment; pre- and post-test questionnaires were used to measure knowledge.

**Results:**

Each of the 36 participants finished both assessments. MEND scores improved statistically significantly in all four departments (*p* = 0.003–0.043), falling from a mean of 26.88 (SD = 6.23) at baseline to 16.97 (SD = 1.06) at 6 weeks (*p* < 0.0001). During the second assessment, no dangerous postures were noted. Recognition of the etiology of carpal tunnel syndrome rose from 10 to 95%, while knowledge of the purpose of ergonomics improved from 49 to 92%.

**Conclusion:**

Clinical postgraduate dentistry students’ chairside ergonomic behavior and understanding significantly and consistently improved because of an organized, interprofessionally presented ergonomic module. It is necessary to incorporate such a program from the very beginning of clinical training.

## 1 Introduction

It has long been known that the physical demands of dental work can lead to occupational injuries. To access the oral cavity, practitioners frequently adopt restricted, asymmetric postures. Over time, this mechanical loading results in cumulative trauma disorders (CTDs), which impact the head, neck, and shoulders (1–3). The wider cost is significant: occupational health issues related to dentistry practice are becoming more common and have a quantifiable detrimental effect on practitioners’ quality of life (2, 3). By matching physical demands with the practitioner’s capabilities, ergonomics—the science of tailoring the workplace to the worker—offers a workable foundation for reducing this stress (3).

In Egypt,^1^ Poland,^4^ South Africa,^5^ and across broader international reviews,^6^ dental professionals and students have been shown to have a higher incidence of work-related musculoskeletal diseases (WMSDs) (1, 4–6). Occasional back or neck pain may be written off as normal, but if symptoms persist and are not treated, cumulative damage can lead to a handicap that ends one’s career. According to research, untreated MSDs can cause one-third of early retirements in dentistry as well as job loss and income loss (1, 7).

Globally, the prevalence of work-related musculoskeletal disorders (WRMSDs) among dental professionals is staggeringly high, with estimates ranging between 64 and 93% (8, 9). In the Indian context, literature reports that the prevalence spans from 19.4% to as high as 100%, with the neck (57.6%) and lower back (up to 68.1%) identified as the most chronically affected anatomical sites (9–12). Within dental specialties, the ergonomic burden varies significantly. Restorative/Conservative dentistry, Endodontics, and Prosthodontics consistently report the highest rates of musculoskeletal discomfort (12, 13). This is primarily attributed to the prolonged static postures, micro-movements, and restricted operational angles required during complex crown preparations, root canal treatments, and long-duration restorative procedures (12, 13).

In India, the risk begins at an early stage of the dental training. According to the Dental Council of India (DCI) norms, dental students enter patient care from the third year of their undergraduate program, performing scaling, restorations, extractions, and prosthetic procedures without a chairside assistant and managing all auxiliary tasks independently (8). All auxiliary duties—maintaining sterile work areas, mixing dental materials, and processing radiographs—are performed by the student alone. The postural habits formed during this formative period may set the trajectory for injuries across an entire career. Significantly, this danger increases even more at the postgraduate level, where access to constrained anatomical areas creates additional restrictions on operator posture, operations are more technically difficult, and operating times are longer.

Despite this acknowledged risk, ergonomic education in dentistry curriculum is still uneven and sometimes limited to short didactic sessions without interprofessional participation, self-assessment tools, or practical reinforcement (7, 10). Using the complementary skills of physiotherapists, orthopaedic surgeons, occupational therapists, and dental faculty, interprofessional education (IPE), where students and professionals from various health disciplines learn together to achieve shared outcomes, offers a promising way to deliver ergonomic content more effectively.

Considering this, the goal of the current study was to create and assess a structured interprofessional participatory ergonomic training module for postgraduate dentistry students across clinical departments. The study specifically aimed to identify ergonomic knowledge gaps and inform module content through a preliminary needs assessment; develop the module through organized interprofessional collaboration spanning occupational therapy, orthopaedic surgery, physiotherapy, and dental expertise; and assess the module’s effectiveness on chairside ergonomic behavior and knowledge using pre- and post-test questionnaires and an unannounced photographic assessment scored with the validated MEND (Meenakshi’s Ergonomic Needs for Dentistry) tool.

## 2 Materials and methods

### 2.1 Study design

To investigate the connection between educational intervention and modifications in ergonomic behavior and knowledge, this study used a quasi-experimental single-group pre-post design. The intact nature of the postgraduate cohort—a single, yearly enrolled group—precluded randomization, even though a randomized controlled trial would provide stronger causal inference (7, 8). The quasi-experimental design is well-established and suitable for educational intervention research of this kind.

### 2.2 Ethical approval

Before participants were recruited, the JSS Academy of Higher Education and Research’s Institutional Review Board gave its ethical approval in accordance with the Declaration of Helsinki with JSSDCH IEC Research Protocol No:01/2024. Prior to enrollment, each subject gave written informed permission. The students were informed that their academic standing would not be impacted by their nonparticipation, and participation was entirely voluntary. Individual data confidentiality was upheld throughout the trial, and only group-level results were disclosed.

### 2.3 Setting

The study was carried out at the JSS Dental College and Hospital, a sizable academic dentistry tertiary care facility connected to the JSS Academy of Higher Education and Research in Mysore, Karnataka, India. With DCI-regulated seat allotment by department per academic year, the institution offers postgraduate studies in a variety of dental specializations.

### 2.4 Participants and eligibility

Postgraduate dentistry students in clinical departments - defined as those where students regularly provide direct patient care involving chairside operational procedures - were the study’s target population. The key outcome measure, chairside ergonomic posture as measured by the MEND tool, is only relevant and scoreable in practitioners who are actively involved in patient treatment. This eligibility condition was essential to research design. Because the ergonomic exposures and assessment contexts in non-clinical or mostly laboratory-based postgraduate programmes (such as Oral Pathology, Oral Microbiology, and Public Health Dentistry) differ significantly from those in clinical practice, these students were omitted.

Prosthodontics, Periodontics, Oral Surgery, Conservative Dentistry, and Endodontics were the five clinical departments that satisfied the eligibility requirements (Table 1). This is a census of the specified target population rather than a convenience subsample because all 36 postgraduate students enrolled in these departments throughout the study period were invited to participate. The within-group selection bias was removed with this method. The DCI-regulated intake capacity determines the total annual enrolment across these clinical postgraduate programmes; therefore, the figure of 36 reflects a structurally constrained, institutionally complete cohort rather than an arbitrary researcher choice. All 36 participants consented and completed both assessments, yielding a 100% retention rate.

**Table 1:** Participant eligibility criteria.

Criterion	Detail	Rationale
Inclusion	Postgraduate students enrolled in a clinical department (Prosthodontics, Periodontics, Oral Surgery, Conservative Dentistry and Endodontics)	Direct chair-side patient care is prerequisite for valid MEND tool assessment
Inclusion	Active direct patient care responsibilities during the study period	Ensures scorable ergonomic postures during unannounced photographic evaluations
Exclusion	Postgraduate students in non-clinical or predominantly laboratory-based specialties (e.g., Oral Pathology and Microbiology; Public Health Dentistry)	MEND tool items require live operative procedures; these students lack the requisite chair-side exposure
Exclusion	Students on approved leave for two or more consecutive weeks during the six-week program	Insufficient exposure to intervention content to permit valid pre–post comparison

### 2.5 The Interprofessional education team

The IPE team comprised the principal investigator (a prosthodontics specialist), a physiotherapist, an orthopaedic surgeon, an occupational therapist, institutional leadership, and dental equipment vendors. Their collective expertise spanned musculoskeletal assessment, clinical biomechanics, workstation ergonomics, and dental-equipment specifications. All team members actively contributed to module content development, instructional material preparation, photographic scoring, and structured feedback delivery. Regular team meetings were held throughout the six-week program, although scheduling conflicts on two occasions limited the breadth of IPE team feedback during those sessions, a limitation discussed further in Section 5.

### 2.6 Preliminary needs assessment

Prior to module development, a self-administered questionnaire was distributed to 103 dental professionals across three stakeholder groups: dental practitioners (41%), postgraduate and undergraduate students (34%), and faculty members (25%). Mapping the frequency of ergonomic issues, identifying knowledge gaps, and making sure that the module’s content was grounded in actual clinical concerns rather than being created in a vacuum were the objectives. The themes, breadth of coverage, and structure of the practical tasks in the module were all directly influenced by the assessment results.

### 2.7 Intervention: the six-week ergonomic module

The program was delivered over six consecutive weeks as follows:

- Week 1: Baseline assessment: Participants answered a validated, self-administered pretest questionnaire on their typical weekly clinical hours, non-dental physical activity, duration of any WMSD symptoms, and demographics.
- Week 2: During a led group session, students expressed concerns about their job activities and suggested ergonomic solutions. This was followed by the first photographic assessment and participatory discussion. The IPE team improved the modules using the knowledge gained from this conversation. During the regular clinical activities, the primary investigator took an unexpected picture of each student’s chairside posture.
- Week 3—Module delivery: The ergonomic module was presented using structured PowerPoint slides. The topics included seated versus standing postures, the biomechanical basis of cumulative trauma disorders, dental workstation design, the value of interpatient stretching exercises, and the use of ergonomic aids (e.g., magnification loupes, saddle chairs, and adjustable units). Students completed a self-assessment exercise using their Week 2 photographs scored with the MEND tool and submitted it via Google Form.
- Weeks 4 and 5—Ergonomic practice and environmental modification: Interactive sessions addressed specific ergonomic solutions, workstation and environmental adjustments, and chair-side stretching routines. Written handouts with illustrated stretching guides were distributed. A second unannounced photograph was taken during the routine clinical examination.
- Week 6—Post-test and second photographic self-assessment: The post-test questionnaire was administered via Google Forms, covering perceived knowledge, current use of ergonomic strategies, perceived value of the training, and open-ended reflective questions were administered. The students also self-assessed their Week 5 photographs using MEND.

### 2.8 The MEND tool

The Principal Investigator created the validated 16-item MEND (Meenakshi’s Ergonomic Needs for Dentistry) assessment to evaluate ergonomic posture, particularly in the setting of dental practice. The Rapid Upper Limb evaluation (RULA) and the Rapid Entire Body Assessment (REBA), (14) two fundamental, internationally acknowledged ergonomic postural evaluation frameworks, served as the conceptual underpinning for the development and adaptation of the MEND tool. While RULA and REBA offer generalized industrial postural screening, the MEND tool explicitly translates these principles to capture fine-grained dental workplace dynamics, such as operator-to-patient mouth distance, unique instrument grasps/fulcrums, and headrest-dental light configurations. Each of the 16 items, covering parameters including operator chair height, leg angle, patient chair positioning, operator-to-patient mouth distance, upper and lower arm positioning, wrist position, neck and trunk alignment, and use of rests, grasps, and fulcrums, was rated on a three-point scale: acceptable, compromised, or harmful. The final score was expressed as a percentage on a 0–100 scale, where a higher value indicated a greater proportion of compromised or harmful postures; therefore, a reduction in the score between evaluations denoted a genuine ergonomic improvement.

MEND is not an adaptation of any previously published instrument; rather, it is a unique observational tool created by the Principal Investigator(copyright registration number-SW19858/24). Numerous observational and photographic tools for evaluating dental ergonomics have been developed including the Modified Dental Operator Posture Assessment Instrument (15), the Standardized Photometric Assessment Method (16), the Dental Ergonomic Assessment (17), the Competence Assessment of Dental Ergonomic Posture (18), the Posture Assessment Instrument (19), and the Posture Assessment Criteria (20). Importantly, none of these tools has been validated or used in the context of Indian postgraduate dental education, where the clinical procedures are more technically complex, operating times are longer, and students work without chairside assistance. MEND was created to fill this void by measuring parameters relevant to this clinical setting such as operator-to-patient mouth distance, instrument grasps and fulcrums, and headrest-dental light configurations.

The MEND tool was validated for content validity and reliability by seven external faculty members with expertise in dental ergonomics and occupational health, yielding a Cronbach’s *α* of 0.79 (indicating good internal consistency). The 22-item module evaluation instrument was validated by seven subject-matter experts (Cronbach’s *α* = 0.97, indicating excellent reliability). Digital delivery photographs submitted and scored via Google Form facilitated blinded, independent scoring by the principal investigator and IPE team, maintaining high inter-rater reliability.

### 2.9 Outcome measures

Three dependent variables were assessed.

- Ergonomic behavior: MEND scores from Week 2 (1st evaluation) and Week 5–6 (2nd evaluation) photographs were compared within participants and within departments.
- Ergonomic knowledge: Correct response rates on the 24-item pre- and post-test questionnaires.
- Perceived value of ergonomics: Thematic analysis of open-ended post-test responses regarding students’ attitudes toward ergonomics and the educational module.

### 2.10 Statistical analysis

Given the non-normal distribution of the MEND scores (confirmed by the Shapiro–Wilk test), the Wilcoxon signed-rank test was used to compare the first and second MEND scores within the overall group and within each department. Statistical significance was set at *p* < 0.05. Data were analyzed using SPSS version 26.0 (IBM Corp., Armonk, NY, USA). Descriptive statistics (mean and standard deviation) were calculated for MEND scores and questionnaire responses. Qualitative data from open-ended responses were thematically analyzed by the principal investigator (PI).

## 3 Results

### 3.1 Needs assessment

The preliminary needs assessment surveyed 103 dental professionals across three groups: 41 dental practitioners (42%), 34 students (34%), and 25 faculty members (25%). The key findings are summarized in Table 2. The majority (96%) were right-handed. Neck pain was reported ‘sometimes’ during work by 47% of respondents, and back pain by 44% of respondents. 57% of participants stated that their daily activities were impacted by musculoskeletal issues, and 94% of individuals thought that dentistry made these issues worse. There was broad agreement that microbreaks are crucial (87%) and that the curriculum should incorporate ergonomic concepts (92%). Despite this knowledge, 65% had never tried chair-side workouts, and just 14% regularly used ergonomic postures in their daily lives, with 50% citing difficulty or distraction as implementation hurdles. These results immediately influenced module content priorities and validated significant unmet educational gaps.

**Table 2:** Summary of needs assessment findings (*n* = 103).

Finding	Proportion (%)
Right-handed practitioners	96
General practitioners	45
Report neck pain ‘sometimes’ during work	47
Report back pain ‘sometimes’ during work	44
Believe dentistry aggravates MSK problems	94
MSK problems affect daily activities	57
Agree ergonomics should feature in curriculum	92
Agree on importance of microbreaks	87
Never used chair-side exercises	65
Define ergonomics correctly	72
Consistently apply ergonomic postures	14

### 3.2 Participant demographics

Among the 36 postgraduate participants, Conservative Dentistry and Endodontics comprised the largest departmental group (38%, *n* = 14), followed by Periodontics (31%, *n* = 11 participants). Prosthodontics and Oral Surgery each contributed 16% (*n* = 6 per department). Females comprised 75% of the cohort (*n* = 27), and males comprised 25% (*n* = 9). All 36 students completed both the pre- and post-test assessments and photographic evaluations, yielding a retention rate of 100%.

### 3.3 Instrument reliability

The 22-item module evaluation tool returned a Cronbach’s *α* of 0.97, confirming excellent internal consistency. The MEND tool achieved a Cronbach’s α of 0.79 across seven external faculty validators, indicating its good reliability. These values indicate that both instruments measured their respective constructs with acceptable consistency and were appropriate for use in an interventional study of this magnitude.

### 3.4 Pre-test ergonomic knowledge

Baseline questionnaire performance varied considerably across the 24 items (Table 3). All 36 participants (100%) correctly identified the general factors contributing to MSDs; however, only 10% correctly identified the role of tendons in carpal tunnel syndrome (CTS), and only 8% correctly identified conditions that were not caused by frequent reaching and twisting in awkward positions. While 91.7% understood the definition of ergonomics, 52.8% correctly identified its goal and best ergonomic practices for dental practitioners. The correct identification of the first symptom of CTS was 36%. These baseline deficits confirmed that students’ theoretical ergonomic knowledge was substantially incomplete, thus validating the need for an educational module.

**Table 3:** Pre-test ergonomic knowledge among clinical postgraduate dental students (*n* = 36).

Parameter	Correct (yes)		Incorrect (no)	
	*N*	%	*N*	%
What is ergonomics?	33	91.7	3	8.3
The goal of ergonomics is to:	19	52.8	17	47.2
Ergonomics is defined as the interaction of individuals and jobs; best practices increase comfort, productivity, and safety	19	52.8	17	47.2
Factors contributing to musculoskeletal disorders (MSDs)	36	100.0	0	0.0
First symptom of carpal tunnel syndrome (CTS)	14	38.9	22	61.1
Role of tendons in CTS	4	11.1	32	88.9
The prevention of CTS can be accomplished by:	27	75.0	9	25.0
Ideal working position for a dental operator	12	33.3	24	66.7
Static body position is better practice than actively changing positions (False)	10	27.8	26	72.2
Best ergonomic tools and practices for dental practitioners	19	52.8	17	47.2
Physical challenges faced by dental professionals	27	75.0	9	25.0
Conditions NOT caused by frequent reaching and twisting in awkward positions	3	8.3	33	91.7
Deviation from the neutral position includes the following	15	41.7	21	58.3
Repetitive motion, overflexion, and overextension of the wrist increase the risk of	11	30.6	25	69.4
To relieve stress on the back, neck, and shoulders, a dental operator can:	15	41.7	21	58.3
To prevent eyestrain, the dental operator should:	16	44.4	20	55.6
Ambidextrous gloves can place excessive tension on the	9	25.0	27	75.0
During procedures, frequently used items should include:	11	30.6	25	69.4
The risk of cumulative trauma disorders (CTDs) can increase with:	9	25.0	27	75.0
The body mechanics for the chairside dental operator include:	17	47.2	19	52.8
Optimal operator positioning includes the following:	17	47.2	19	52.8
Ergonomic chairside suggestions include the following:	6	16.7	30	83.3
How often should you change your body position while working?	12	33.3	24	66.7
Which part of the back should the chair provide support?	26	72.2	10	27.8

*N* and % denote the number and percentage of participants who answered correctly (Yes) or incorrectly (No) for each item.

### 3.5 Photographic ergonomic assessment—first evaluation (week 2)

Scoring of the baseline photographs revealed a predominantly compromised ergonomic profile: 72% of the MEND observations across all 16 items were rated as compromised, 28% as harmful, and none were rated as acceptable. Female participants accounted for seven harmful and 17 compromised observations, and male participants contributed two harmful and six compromised observations, which was consistent with the sex composition of the cohort.

The leg angle (63% compromised), operator-to-patient mouth distance (63% compromised), and upper-arm positioning (47% compromised) were the most consistently affected parameters. The neck and trunk/spine showed particularly high rates of harmful postures (25 and 16%, respectively). Feet resting flat on the floor was the most consistently acceptable parameter (69%), suggesting that lower limb positioning was less affected than upper body and spinal posture. These findings identify priority areas for targeted instruction.

### 3.6 Photographic ergonomic assessment—second evaluation (week 5–6)

By the second evaluation, the ergonomic profile improved substantially across all parameters. Acceptable observations accounted for 44% of the MEND ratings, and critically, no harmful postures were recorded; all residual compromises (56%) were at the intermediate level. Feet positioning, the operator’s chair, and the patient’s chair had 100% acceptability. Leg positioning, pelvis, wrist, instrument rests, grasps and fulcrums, neck, shoulder, upper arms, headrest, trunk/spine, and dental light positioning showed residual compromise rates ranging from 6 to 13%, identifying them as targets for continued reinforcement beyond the initial six-week program.

### 3.7 MEND score comparison by department

The Wilcoxon signed-rank test confirmed statistically significant reductions in MEND scores across all departments (Table 4). Overall, the mean decreased from 26.88 (SD = 6.23) at baseline to 16.97 (SD = 1.06) at 6 weeks (*p* < 0.0001). Regardless of the participants’ initial posture, the module induced consistent ergonomic behavior, as evidenced by the standard deviation lowering from 6.23 to 1.06, indicating that the scores improved and converged.

**Table 4:** MEND tool scores at first and second evaluations by department (Wilcoxon signed-rank test).

Department	Evaluation	Mean score	SD	*p*-value	Sig.	*n*
Conservative and endodontics	First MEND	24.00	6.41	0.003	^*^	14
	Second MEND	17.08	0.99			
Oral surgery	First MEND	28.20	7.56	0.043	^*^	6
	Second MEND	16.80	0.84			
Periodontics	First MEND	26.90	5.09	0.005	^*^	11
	Second MEND	17.20	1.32			
Prosthodontics	First MEND	32.40	2.61	0.042	^*^	6
	Second MEND	16.40	0.89			
Overall	First MEND	26.88	6.23	<0.0001	^*^	36
	Second MEND	16.97	1.06			

SD, standard deviation. The MEND scores range from 0 to 100; a lower score denotes fewer compromised or harmful postures (i.e., better ergonomics). ^*^p < 0.05 (statistically significant). *p*-value reported only for the paired comparison between the 1st and 2nd MEND within each department.

Conservative dentistry and endodontics had the lowest baseline mean score (24.00, SD = 6.41), whereas prosthodontics had the highest (32.40, SD = 2.61). Despite this discrepancy, at the end evaluation, all departments had significantly improved (*p* = 0.003–0.043), indicating that the module’s advantages were applicable to a variety of clinical settings.

### 3.8 Post-test knowledge gains

Post-test questionnaire data confirmed substantial gains in ergonomics knowledge (Table 5). The correct identification of the goal of ergonomics increased from 49 to 92% (+43 points). Recognition of the first symptom of CTS increased from 36 to 64% (+28 points), knowledge of CTS prevention from 69 to 97% (+28 points), and correct identification of the role of tendons in CTS from 10 to 95% (+85 points). Open-ended post-test responses indicated that students experienced a qualitative shift in their understanding, from viewing ergonomics as an abstract classroom concept to recognizing its direct relevance to their clinical performance, physical health, and career longevity in the field.

**Table 5:** Selected pre- and post-test knowledge outcomes (*n* = 36).

Parameter	Pre-test %	Post-test %	Change
Goal of ergonomics correctly identified	49	92	+43%
First symptom of CTS correctly identified	36	64	+28%
Prevention of CTS correctly identified	69	97	+28%
Role of tendons in CTS correctly identified	10	95	+85%

## 4 Discussion

This study showed that chairside ergonomic behavior and ergonomic knowledge among postgraduate dentistry students in clinical settings can be improved statistically significantly and clinically meaningfully by an organized, interprofessionally presented ergonomic module. These results are consistent with and add to the body of data from similar interventional studies (7, 8, 10), but they also make a few additions that set this work apart from other research.

### 4.1 Interprofessional collaboration as a delivery model

In addition to the lead investigator from the dental department, the participation of physiotherapists, orthopaedic surgeons, occupational therapists, and equipment vendors gave students a multifaceted understanding of ergonomic risk that is not possible with a single-discipline approach (7, 10). From musculoskeletal biomechanics and injury rehabilitation to workstation specifications and equipment adjustment, each team member provided domain-specific knowledge that together form the whole ergonomic picture pertinent to dental practice. In addition to maintaining high inter-rater reliability and reducing the time burden on assessors and students, the use of digital photos submitted via Google Forms for self-assessment and IPE feedback showed that interprofessional ergonomic education can be effectively delivered in a busy academic dental setting.

### 4.2 The MEND tool as a reflective learning and assessment tool

A key strength of this study is the use of the validated MEND tool for both summative assessment and formative self-reflection. Its 16-item observational structure provided sufficient granularity to identify specific postural deficits at baseline—namely, harmful neck and trunk/spine postures affecting 25 and 16% of observations, respectively—and to track targeted improvement over 6 weeks. The near elimination of harmful ratings by the second evaluation (from 28 to 0%) is a clinically important finding because harmful postures represent the highest-risk ergonomic category and, if sustained, are most directly associated with cumulative injury. The narrowing of the MEND score standard deviations (6.23–1.06) indicates that the module did not merely improve average performance but produced consistent, uniform improvement across the cohort, a hallmark of effective instructional design (7).

### 4.3 Knowledge gains and attitudinal change

Despite prior exposure to ergonomic concepts during their undergraduate training, participants entered the study with substantial knowledge gaps; most strikingly, only 10% could correctly identify the role of tendons in CTS, and only 8% could correctly identify conditions not caused by repetitive reaching and twisting. This supports the findings of Mulimani et al. (21) and Beach et al. (22) who have been cited in the literature, that dental programmes provide basic ergonomic instruction but fail to equip students with the full spectrum of biomechanical and preventive knowledge needed for sustained safe practice (7, 10). The post-test gain of 85 percentage points on tendon-in-CTS knowledge and 43 points on the goal of ergonomics demonstrates that the module effectively addressed these gaps.

Beyond declarative knowledge, open-ended responses revealed a deeper shift: students moved from perceiving ergonomics as a peripheral curricular obligation to recognizing it as a determinant of their physical health, clinical performance, and longevity in the profession. This attitudinal change is consistent with Kirkpatrick’s Level 2 (Learning) and Level 3 (Behavior) outcomes and aligns with the pre–post improvements in MEND scores, indicating that knowledge gains were translated into behavioral change within the study period (6).

### 4.4 Departmental variation and clinical specificity

The variation in baseline MEND scores across departments warrant specific consideration. Prosthodontics recorded the highest initial ergonomic burden (mean 32.40), consistent with the postural demands of prosthodontic procedures, including extended static positioning, high-precision restorative and impressioning tasks, and frequent access to constrained operating angles. Conservative Dentistry and Endodontics had the lowest baseline (24.00), reflecting slightly greater flexibility in patient and operator positioning during restorative procedures. Oral Surgery and Periodontics occupied intermediate positions.

Critically, all departments achieved significant improvement by the final evaluation, with post-intervention means converging to a narrow range (16.40–17.20). This convergence suggests that a single, well-designed module can effectively serve clinically diverse populations, regardless of specialty-specific baseline ergonomic burden. This also indicates that the MEND tool was sensitive enough to detect improvements across different procedural contexts, which is an important validation of the instrument’s clinical breadth. Specialty-specific add-on modules, particularly for high-burden disciplines such as prosthodontics, would be a logical next step in addressing residual specialty-specific risks.

### 4.5 Institutional impact

Additionally, the ergonomically designed dental operator’s chair developed through the project was granted a patent on September 2, 2024 (patent no 549500- Ergonomically designed operators chair for dental practitioners to perform dental procedures) Although there are currently no published stand-alone clinical trials or long-term studies assessing this particular patented chair design, its engineering is firmly rooted in the body of research confirming the effectiveness of customized seating interventions. In comparison to conventional flat operator stools, previous research on specialized dental seating—such as saddle chairs and chairs with dynamic lumbar support systems—has demonstrated structural efficacy in lowering lower back strain, minimizing unnatural pelvic tilting, and reducing intradiscal pressure. A planned course for our team’s future study is the independent clinical assessment of our innovative chair.

### 4.6 Limitations

Several limitations of this study require transparent acknowledgement and should inform the interpretation of the findings.

First, the biggest drawback was the lack of a contemporaneous control group. It is impossible to conclusively link all reported benefits to the instructional modules in the absence of a matched comparison cohort that received no intervention (or an alternative intervention). It is impossible to completely rule out the Hawthorne effect as a contributing reason to the gains in MEND scores, as individuals alter their behavior when they are aware that they are being watched and photographed. To establish more robust causal inferences, future research should include a parallel cohort from similar institutions or a waiting control group.

Second, external generalizability is limited by the sample of 36 students from a single institution, even though they make up the entire eligible clinical postgraduate cohort. Smaller or rural dental institutions with varied resource profiles, curriculum frameworks, and patient demographics might not have the same institutional setting as a large tertiary academic dentistry facility in urban Karnataka. To prove generalizability, multi-site replication is necessary.

Third, findings on the long-term sustainability of ergonomic behavioral changes were not possible due to the six-week evaluation window. It is unclear if the benefits seen here will continue after the study period’s accountability structure—regular photos, IPE comments, and reflective self-evaluation—is eliminated. To find out if improvements are maintained, need to be reinforced, or deteriorate over time, longitudinal follow-up at 3, 6, and 12 months would be crucial.

Fourth, residual observer variability in photographic scoring cannot be completely ruled out, despite the MEND tool’s strong reliability (Cronbach’s *α* = 0.79). Photographs may not accurately depict students’ typical posture patterns because they only record a single time during a clinical session. To give a more complete picture, future evaluations might add wearable sensor-based posture monitoring or continuous observational recordings to photographic assessments.

Fifth, scheduling conflicts within the IPE team during the two sessions of the program meant that feedback from some team members was curtailed. A phased or extended delivery model with built-in scheduling redundancy could reduce this risk in future studies.

Sixth, the study did not include undergraduate dental students, who arguably carry the greatest risk given their earlier and less supervised clinical exposure. Extending this program to include the undergraduate curriculum, ideally from the point of first patient contact in the third year, would represent the most impactful application of this model.

Anthropometric information such as height, weight, and body mass index was not gathered for this investigation. Individual reactivity to ergonomic training, load distribution at the operatory, and baseline posture may all be influenced by physical attributes. The current design is limited by their absence. To account for individual physical heterogeneity in ergonomic behavior, future research should include anthropometric profile and investigate its link with baseline MEND scores and intervention-driven change scores.

## 5 Conclusion

Musculoskeletal disorders in dentistry are not inevitable; they are, to a meaningful extent, preventable, however they continue to accumulate as each cohort of graduates enters the practice with insufficient ergonomic preparation (4). This study demonstrated that a structured, interprofessionally delivered ergonomic module produced significant, consistent, and broadly applicable improvements in chairside ergonomic behavior and knowledge among postgraduate clinical dental students.

The combination of collaborative IPE delivery, unannounced photographic assessment with the validated MEND tool, and guided self-reflection proved particularly effective, eliminating all harmful postures by the final evaluation and reducing the mean MEND scores by 37%. The model is practically sustainable since its digital components lessen the load of assessment for both teachers and students, and it can be taught within a regular academic calendar without the need for specialized equipment.

Most importantly, ergonomic education needs to start before employees develop bad behaviors. It has the potential to prevent injury accumulation, prolong career longevity, lower the financial costs of work-related disability, and ultimately improve the quality of care provided to patients by practitioners who are physically fit and at ease in their workplace by incorporating a program of this kind from the very beginning of clinical training, including mannequin training in the pre-clinical years.

## Data Availability

The original contributions presented in the study are included in the article/supplementary material, further inquiries can be directed to the corresponding author.
